# Dynamic Phosphoproteome Analysis of Seedling Leaves in *Brachypodium distachyon* L. Reveals Central Phosphorylated Proteins Involved in the Drought Stress Response

**DOI:** 10.1038/srep35280

**Published:** 2016-10-17

**Authors:** Lin-Lin Yuan, Ming Zhang, Xing Yan, Yan-Wei Bian, Shou-Min Zhen, Yue-Ming Yan

**Affiliations:** 1 College of Life Science, Capital Normal University, 100048 Beijing, China; 2 College of Life Science, Heze University, 274015 Shandong, China

## Abstract

Drought stress is a major abiotic stress affecting plant growth and development. In this study, we performed the first dynamic phosphoproteome analysis of *Brachypodium distachyon* L. seedling leaves under drought stress for different times. A total of 4924 phosphopeptides, contained 6362 phosphosites belonging to 2748 phosphoproteins. Rigorous standards were imposed to screen 484 phosphorylation sites, representing 442 unique phosphoproteins. Comparative analyses revealed significant changes in phosphorylation levels at 0, 6, and 24 h under drought stress. The most phosphorylated proteins and the highest phosphorylation level occurred at 6 h. Venn analysis showed that the up-regulated phosphopeptides at 6 h were almost two-fold those at 24 h. Motif-X analysis identified the six motifs: [sP], [Rxxs], [LxRxxs], [sxD], [sF], and [TP], among which [LxRxxs] was also previously identified in *B. distachyon*. Results from molecular function and protein-protein interaction analyses suggested that phosphoproteins mainly participate in signal transduction, gene expression, drought response and defense, photosynthesis and energy metabolism, and material transmembrane transport. These phosphoproteins, which showed significant changes in phosphorylation levels, play important roles in signal transduction and material transmembrane transport in response to drought conditions. Our results provide new insights into the molecular mechanism of this plant’s abiotic stress response through phosphorylation modification.

Drought is one of the most important sources of abiotic stress, as it limits growth, development, and yield of field crops. As the world population increases, and available water resources decrease, plant breeders have increasingly focused on the development of drought-tolerant crops with high water-use efficiency^1^. Therefore, it is vital to elucidate the molecular mechanisms of plant responses to drought stress.

Land plants have developed a variety of adaptation and defense strategies in response to internal and external stimuli, particularly for drought stress^2^. Molecular mechanisms in response to drought conditions are elusive, given the complexity of organisms and the limitations of experimental technology. It is commonly accepted that drought responses by plants include osmotic homeostasis or regulation, stress repair and defense, detoxification, and growth inhibition^3^. These processes are likely to follow signal transduction and cell cascades.

Results from recent studies have shown that three signal pathways are involved in drought response and defense: abscisic acid (ABA), reactive oxygen species (ROS), and Ca^2+^ signaling. ABA is an important signal molecule, as it causes the expression of regulation proteins and allows proteins to participate in the response to and defense from various stresses^4^,^5^. Ca^2+^ signaling is also a common signal pathway that responds to adverse signs in eukaryocytes^6^. ROS are involved in the response to oxidative stress, as it serves as a supplementary signal pathway to cope with drought stress^7^,^8^. In signal pathways, the signal processes generally involve protein phosphorylation and dephosphorylation by protein kinases and phosphatases, when subjected to drought stress.

Protein phosphorylation is closely related to a large number of processes, including metabolism, transcription and translation, protein degradation, homeostasis, cellular signaling and communication, and various stress responses^9^,^10^,^11^. Phosphorylation is a reversible and ubiquitous post-translational modification (PTM), which is a dynamic process regulated by kinases and phosphatases. In eukaryotes, phosphorylation occurs more frequently on serine (Ser) and threonine (Thr) than tyrosine (Tyr). Studies have shown that phosphorylation participates in many molecular events and biological processes, such as DNA transcription, stomatal closure^12^, stress response and defense, and energy metabolism^13^.

Recently, large-scale *in vivo* phosphoproteomic analyses have been performed to elucidate diverse response mechanisms in different plant species, such as rice^14^, *Arabidopsis thaliana* L.^15^, soybean^16^, maize^17^, and wheat^18^,^19^,^20^. Moreover, our group characterized the phosphoproteome of seedling leaves of *B. distachyon*^21^. However, the dynamic changes in the phosphoproteome in response to drought remain unknown.

*B. distachyon*, a temperate wild annual grass species typically grown in the Mediterranean and Middle East, has recently been used as a model plant for cereal crops and potential biofuel grasses^22^,^23^. It has a large number of desirable features: a small diploid genome (272 Mbp), short developmental cycle, efficient transgenosis, self-fertilizing and simple nutrient requirements^24^.

In recent years, the rapid development of genome sequencing in *B. distachyon* has facilitated further studies on transcriptomics, proteomics, and phosphoproteomics. In this study, we performed the first *in vivo* dynamic analysis of the phosphoproteome of *B. distachyon* seedling leaves at different time points (0, 6, and 24 h), using TiO_2_ enrichment and LC-MS/MS analysis coupled with label-free quantification. A large number of phosphorylated proteins involved in the drought stress response were identified, providing new information for understanding the molecular mechanisms of plant responses to drought stress.

## Results

### Phenotypic, physiological, and ultrastructural changes in seedling leaves under drought conditions

**Figure 1:**
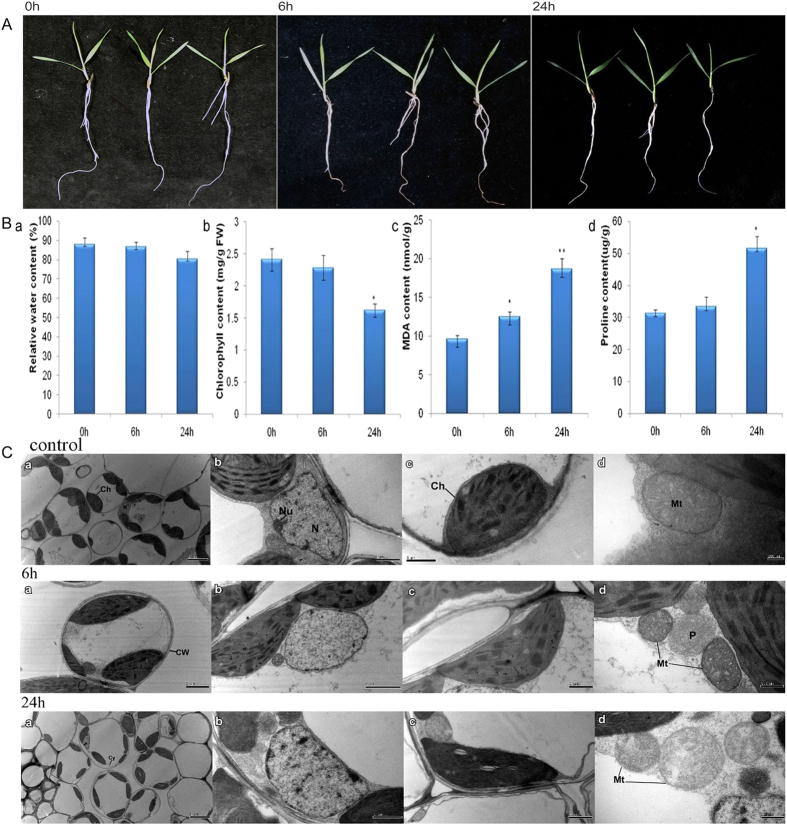
Phenotypic, physiological and ultrastructural changes in Bd21 leaves under 0, 6 and 24 h drought stress. (**A**) Seedling phenotypic changes. (**B**) Leaf physiological changes: a. RWC; b. Chlorophyll content; c. MDA content; d. Proline content. Error bars indicate standard errors of three biological replicates. Statistically significant differences compared to the control were calculated based on an independent Student’s t-tests: *P < 0.05; **P < 0.001. (**C**) Leaf ultrastructural changes observed by TEM. a. Bd21 leaves ultrastructure; b. Nucleolus ultrastructure; c. Chloroplast ultrastructure; d. Mitochondria ultrastructure. Ch: Chloroplast; CW: Cell wall; Cy: Cytomembrane; Nu: Nucleolus; N, Nucleus; Mt: Mitochondria; P: Peroxysome.

The dynamic changes in phenotypes and some important physiological indices of *Brachypodium distachyon* 21 (Bd21) seedling leaves under different drought conditions are shown in Figs 1 and S1. With the intensification of drought stress, leaves and roots displayed more obvious changes. At 24 h, leaves had a slight curl, while roots became thin compared to the control group. There were no significant differences at 6 h (Fig. 1A). Drought stress resulted in significant changes in the physiological index of seedling leaves (Figs S1 and1B). The results from five different periods (0, 6, 12, 24, and 48 h) showed that relative water content (RWC) and total chlorophyll content for the 20% PEG 6000 treatment displayed a continuous declining trend. In contrast, the levels of malondialdehyde (MDA) and proline (Pro) content continuously increased in response to drought stress. The 24 h treatment was a critical turning point, while 6 h was a transition period. Compared to the control group (0 h), MDA exhibited a significant (*p* < 0.05) and highly significant (*p* < 0.01) increase at 6 h (1.299-fold) and 24 h (1.939-fold), respectively. Proline content was significantly increased by 1.646-fold at 24 h (*p* < 0.05).

To reduce experimental error, unified flag leaves of *B. distachyon* were selected for transmission electron microscope (TEM) observation. The ultrastructural changes between the non-stress and drought stress treatments of Bd21 seedling leaves are shown in Fig. 1C. The flag leaves in the control group had normal whole mesophyll cells and clear subcellular structures. Treatment with 20% PEG resulted in different degrees of damage to parts of the mesophyll cells and some organelles, including clear plasmolysis of the cell wall, misshapen chloroplasts and nucleolus, particularly at 24 h (Fig. 1C). With disrupted thylakoids, the number of starch granules increased, mitochondria became swollen, and the color faded. These results demonstrate that drought stress has significant effects on leaf ultrastructure.

### Phosphoproteins identification and locations of phosphorylated sites

According to the dynamic changes in plant phenotype and leaf physiological traits under drought stress, leaves from three key time points (0, 6, and 24 h) were collected and used to study dynamic changes in the phosphoproteins based on phosphopeptides enrichment and LC-MS/MS and MaxQuant analyses (Figs S2 and S3; Table S1). All mass spectrometry proteomics data were deposited in the ProteomeXchange Consortium (http://proteomecentral. proteomexchange.org) via the PRIDE partner repository with the dataset identifier PXD003522. A total of 4924 phosphopeptides, containing 6362 phosphosites, were identified and belonged to 2748 phosphoproteins (Fig. S3a; Table S1). Moreover, 3843 (78.05%) phosphopeptides had only one phosphosite while 838 (17.02%) and 199 (4.4%) phosphopeptides contained two and three phosphosites, respectively. In particular, three phosphopeptides had six phosphosites (Fig. S3b). In Fig. S3c, a total of 5403 (84.92%), 932 (14.65%), and 27 (0.42%) phosphorylation sites were screened separately on Ser, Thr, and Tyr. According to the location probability of phosphorylated sites, 4356 (68.47%), 632 (9.93%), 781 (12.28%), and 593 (9.32%) phosphorylated sites belonged to class I (*p* ≥ 0.9), II (0.75 ≤ *p* < 0.9), III (0.5 ≤ *p* < 0.75), and IV (*p* < 0.5), respectively (Fig. S3d).

**Figure 2:**
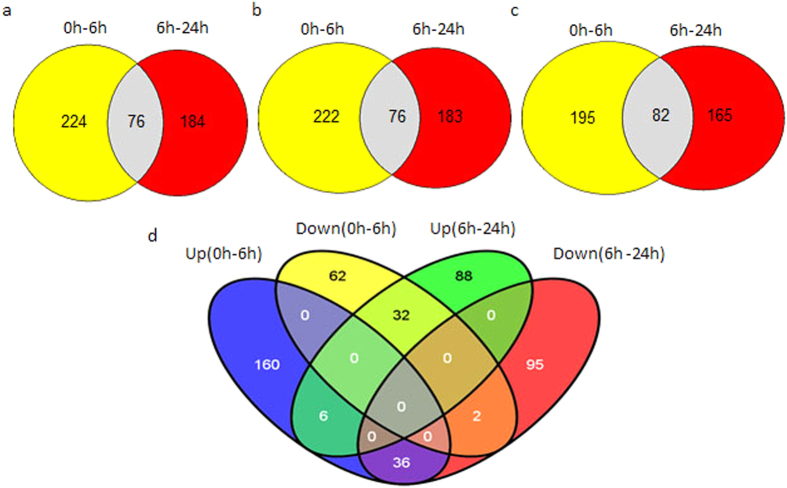
Venn diagram of SCPL phosphorylation data under drought conditions. (**a**) SCPL phosphorylated sites; (**b**) SCPL phosphorylated peptides; (**c**) SCPL phosphoproteins. d. SCPL phosphorylated peptides at different drought stress times.

To increase reliability and accuracy, only phosphorylated sites belonging to the highest phosphorylation site localization probability (*p* ≥ 0.9, class I) were used for further analysis. Phosphopeptides with receivable Student’s t test (*p* < 0.05) consequence, significant intensity changes (≥2-fold), high phosphorylation site localization probability (≥0.9), and phosphorylation site score differences exceeding five were taken into consideration to identify significant changes in phosphorylation level. In the strict limitations screening progress, a total of 484 phosphorylation sites had significant changes in the phosphorylation level (SCPL), and divided into 0–6 h and 6–24 h. Comparisons of phosphorylation sites, phosphopeptides, and phosphorylated proteins are provided in Fig. 2a~c, Tables S2a and S2b, respectively. In two groups, there were 76 phosphorylation sites in common, representing 82 phosphorylated proteins.

A Venn diagram was used to analyze the significantly up-regulated and down-regulated phosphopeptides in both groups (Fig. 2b). The fold change in up-regulated phosphopeptides at 6 h was almost two-fold greater than at 24 h. Six and two phosphopeptides were up-regulated and down-regulated, respectively (Fig. 2d). Proteins involved in stress defense included a stress protein DDR48-like (gi|357163456) and a late embryogenesis abundant protein B19.3 (LEA, gi|721639026).

### Expression pattern analysis of phosphoproteins with SCPL under drought stress

**Figure 3:**
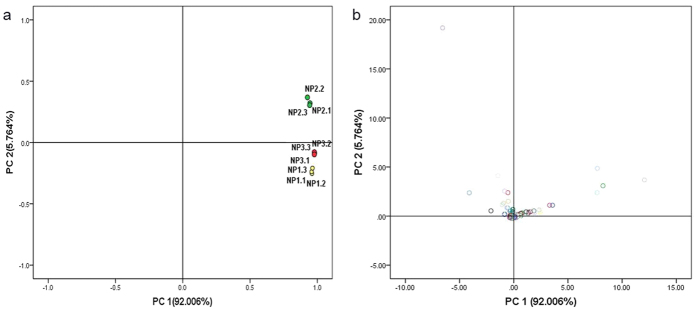
PCA of SCPL phosphoproteins under drought stress. (**a**) PCA of individual proteins samples in seedling leaves. Yellow circles (NP1) represent 0 h, green circles (NP2) represent 6 h and red circles (NP3) represent 24 h. (**b**) PCA of all SCPL phosphoproteins of seedling leaves. NP1, NP2 and NP3 represented the normalized intensities of SCPL phosphopeptides at 0 h, 6 h and 24 h, respectively. NP1-1, NP1-2 and NP1-3 mean the three biological replicates of NP1 (0 h). So does the NP2 and NP3.

Principal component analysis (PCA) is a multivariate method of dimensionality that can be used to identify relationships among variables^25^. The PCA results from 442 SCPL phosphoproteins are shown in Fig. 3. Three groups of biological replication (NP1, NP2, and NP3) showed a high level of reproducibility (Fig. 3a). The treatment groups were significantly different from the control group. The treatment group at 6 h had the greatest differences from the other treatments. Some spots focused on the origin of coordinates and a few had scattered distribution in the periphery (Fig. 3b). Most of the scattered spots belonged to pattern I and pattern II in Fig. S5. Meanwhile, these scattered spots were common spots of 76 phosphorylation sites. These results indicate that Bd21 underwent significant changes in protein phosphorylation levels under drought stress.

To visualize the dynamic changes in the SCPL phosphopeptides, the Euclidean distance similarity metric was used to define the similarity, and hierarchical clusters were assembled using the complete linkage clustering method. Five hierarchical clusters (patterns I–V), corresponding to the different expression patterns, were defined in each period (Fig. S5; Table S3). Pattern I, containing 179 phosphorylation sites, was significantly up-regulated at 6 h, while pattern II, with 106 phosphorylation sites, showed up-regulated expression at 24 h. Furthermore, we classified the function of these two parts phosphorylated proteins in Figs S7a,b. These results further validate the previous PCA analysis, where the 6 h treatment had significantly more SCPL proteins compared to the other treatments.

### Phosphopeptide screening and placement in functional categories

**Figure 4:**
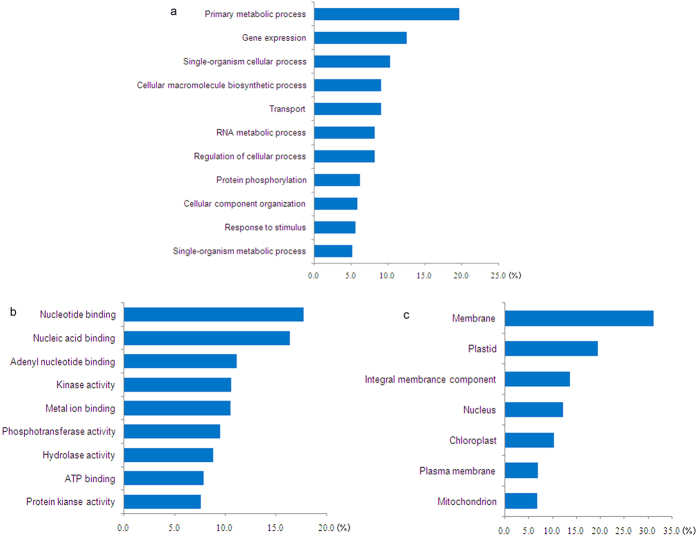
Functional category of SCPL phosphoproteins subjected to drought stress. (**a**) Biological processes. (**b**) Molecular functions. (**c**) Cellular components.

**Figure 5:**
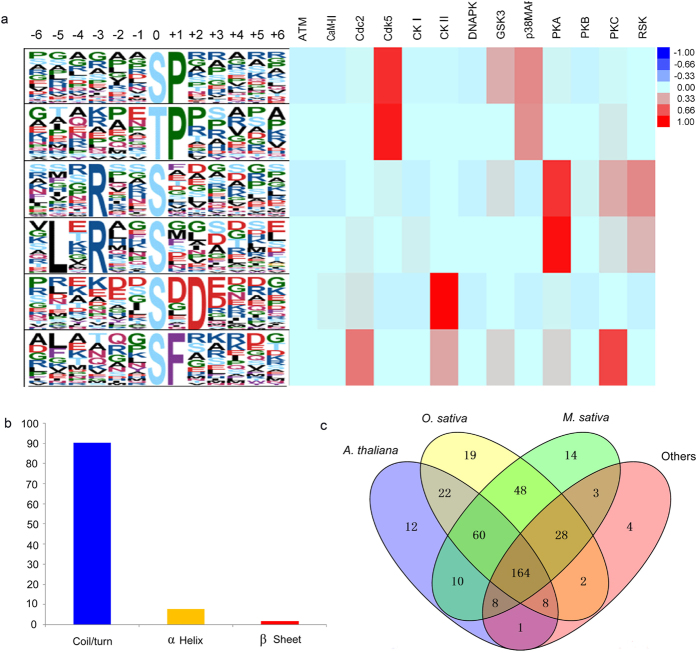
Phosphorylation motif, protein secondary structure surrounding the phosphorylation site and conservation of the significantly changed phosphoproteins under drought stress. (**a**) Significantly enriched phosphoproteins by Motif-X: left part represents amino acids surrounding the identified phosphorylated residues by Motif-X under drought stress; Right part represents phosphorylation of the related kinases according to the score height which are divided into different groups. (**b**) Protein secondary structure surrounding the phosphorylation site, including random coils or turns, α-Helix and β-pleated sheet. (**c**) Conservation analysis of the significant changed phosphoproteins.

All of the identified SCLP proteins were used to predict the functional categories with GO annotation. They were involved in different biological processes, molecular functions, and cellular components (Fig. 4; Table S4). Regarding biological processes, primary metabolic processes accounted for 20% in Fig. 4a. The proportion of gene expression and single-organism cellular process was less than primary metabolic process, respectively. Nucleotide and nucleic acid binding occupied the main part of the molecular functions (Fig. 4b), indicating that nucleoproteins respond to drought stress positively and phosphorylation is one of the coping mechanisms. The cellular components of more than 30% of the phosphoproteins were localized to the membrane (Fig. 4c), indicating that they play a key role in metabolism and some phosphoproteins of water and ion transport were identified in Table 1. Furthermore, three aquaporins (AQPs) were analyzed by 3D structural analyses and sequence alignment in Fig. S6. These AQPs were aquaporin PIP1-5 (IPI1-5, gi|357137703), probable aquaporin IPIP2-7 (IPIP2-7, gi|357159722), and aquaporin NIP2-2 (NIP2-2, gi|357124731). In 3D structural analyses, only PIP1-5 phosphorylation site belonged to the α-helix. Others were on the random coil, which is in agreement with the secondary structure surrounding the phosphorylation site prediction results (Fig. 5b). Most of the SCPL phosphoproteins (nearly 90%) were random coils or turns, 7.6% were α-helixes, and the remaining proteins (1.6%) were β-pleated sheets (Fig. 5b; Table S5b).

Some SCPL proteins identified by Lv *et al.*^26^ under salt stress were also identified under drought stress in this study (Table 2). Their functional classification is provided in Fig. S7c. A total of 51 phosphorylated proteins were identified; 31% of them are related to transcription and translation, and approximately 18% participate in water and ion transport related to osmotic regulation. However, 29% of the proteins are associated with other processes and require further study (Fig. S7c).

### Phosphorylation motif analysis of significantly changed phosphopeptides

Phosphorylation motifs of the phosphoproteins linked to kinase were ascertained by Motif-X and WebLogo online software tools (Fig. 5a; Table S5a). There were six motifs were identified, including five phosphorylation residues were enriched by Ser, (such as [sP], [Rxxs], [LxRxxs], [sxD], and [sF]) and only [TP] motif was enriched by Thr and Pro. Some luxuriant kinases were identified by hierarchical clusters. Both [sP] and [TP] were proline-directed motifs, recognized by cyclin-dependent kinase 5 (Cdk5)^27^. The motifs of [Rxxs] and [LxRxxs] were basic motifs recognized by protein kinase A (PKA)^28^. In addition, motif [sxD] was an acidic motif recognized by casein kinase-II (CKII)^29^. Only motif [sF] was easily recognized by protein kinase C^30^. [LxRxxs] is a newly identified motif in *B. distachyon*. Some key phosphoproteins, including heat shock protein 83 (Hsp 83, gi|357152022), zinc finger CCCH domain-containing protein 13 (gi|357117322), chloride channel protein CLC-c-like (gi|357149553), transcription factor bHLH128-like (gi|357126351), and calmodulin-binding transcription activator 3-like isoform X1 (gi|721658612), were involved in this motif.

### Conservation analysis of phosphoproteins responsive to drought stress

Sequences of the SCPL phosphoproteins were used as queries to BLAST search the phosphoprotein databases constructed based on datasets in the Plant Protein Phosphorylation DataBase (P3DB)^31^, Medicago-Omics Repository (MORE)^32^, and PhosPhAt 4.0^33^. *Oryza. sativa*, *A. thaliana*, *Medicago truncatula*, and others were compared to *B. distachyon* to determine the extent of conservation of phosphoproteins among different plant species. The thresholds were set at a score ≥80, E-value < 1E−10, and identity ≥30% (Fig. 5c; Table S5c). In total, among the 442 phosphoproteins identified in *B. distachyon*, 104 (23.48%), 86 (19.41%), and 49 (11.06%) had phosphorylated orthologs in three, two, and one species, respectively. Only 164 phosphoproteins had phosphorylated orthologs in all four species. A total of 40 (9.02%) phosphoproteins had no phosphorylated orthologs in any of these species.

### Protein–protein interaction (PPI) analysis

To explore the relationships in the PPI network, 442 SCPL proteins identified in the current study were analyzed by STRING. A total of 307 EuKaryotic Orthologous Groups (KOGs) were used to construct the PPI network. To improve the reliability of PPI analysis, the confidence score was set at the highest level (≥0.900). Finally, a complex PPI network was completed through Cytoscape (Fig. S8; Table S6).

Numerous SCPL phosphoproteins were found to participate in transcription and translation of gene expression (Fig. S8). Some of the phosphoproteins belonged to nucleotide or nucleic acid binding phosphorylation proteins, in agreement with the functional categories (Fig. 4b). Moreover, some of the important protein kinases and phosphatases, including abscisic acid-inducible protein kinase (PKAB1, KOG0583), myotonin protein kinase 7–1 (MPK7-1, KOG0660), calcium-dependent protein kinase 26-like (CDPK, KOG0032), and the probable protein phosphatase 2C 47 (PP2C, KOG0698), played a key role in this PPI map, which participated in protein phosphorylation and dephosphorylation under drought stress. The proteins in this map were also involved in signal transduction, water and ion transport, cell structure and division, and photosynthesis and energy metabolism.

### Verification of identified phosphoproteins by Pro-Q diamond staining and tandem mass spectrometry

To enhance the credibility of results, Pro-Q diamond staining and tandem mass spectrometry were used to validate the phosphoproteins identified in this study. The results from Pro-Q diamond and Coomassie brilliant blue (CBB) showed that 33 protein spots were phosphorylated at 6 and 24 h under drought stress (Fig. S4). MALDI-TOF/TOF-MS identification showed that these protein spots represented 24 phosphoproteins, further confirming the results from phosphoproteome analysis (Table S7). Four spots (1, 11, 14, and 24) belonged to three SCPL phosphoproteins: fructose-bisphosphatealdolase (chloroplastic, gi|357157399), ribulosebisphosphate carboxylase small chain PW9 (chloroplastic-like, gi|357146204), and ATPase beta subunit (chloroplast, gi|193075564). These phosphoproteins are related to photosynthesis and energy metabolism.

## Discussion

### Protein kinases/phosphatases participated in signal perception and transduction

**Figure 6:**
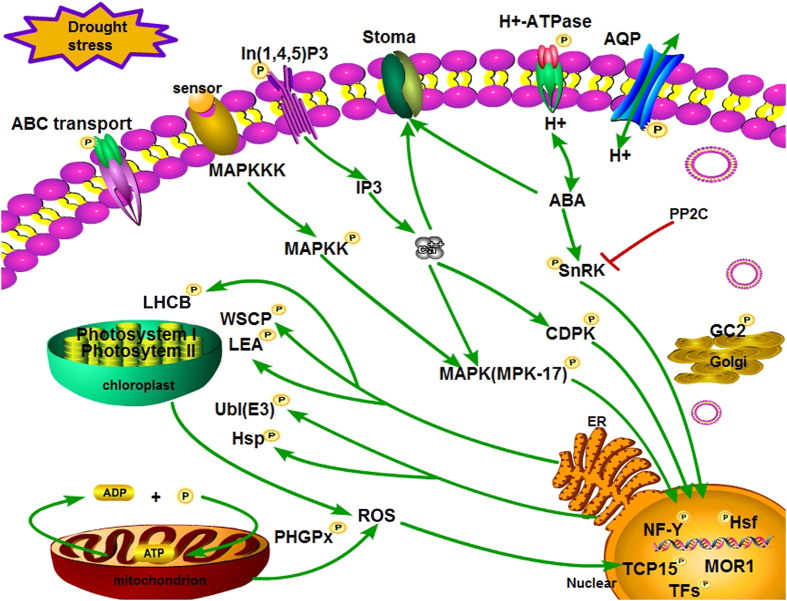
A putative pathway of drought stress responses in Bd21 leaves through phosphorylation modification. The SCPL phosphoproteins identified in our study were used to construct the putative pathway under drought stress. Yellow ball represented phosphoproteins. Red line represented inhibiting effect.

The reversible phosphorylation modification event is regulated by protein kinase and phosphorylase, which could supervise multiple biological processes of organisms^34^. In this study, numerous protein kinases and phosphatases were SCPL, and played key roles in signal transduction (Figs 6 and S8). ABA is a crucial signal molecule in signal transduction responses to various abiotic stresses, including drought stress^35^. PKABA1 (gi|721630009) phosphorylated at Ser622 was identified in this study and found to be significantly up-regulated at 24 h (Table S2b). PKABA1, a member of the sucrose non-fermenting1-related protein kinase (SnRK) subfamily, acts as an intermediate in ABA suppression of GA-responsive gene transcription^36^. Some evidence suggested that over-expression of *TaSnRK2S* could enhance the tolerance of abiotic stressors, such as drought and salt stress, in *A. thaliana*^37^. In addition, protein phosphatase 2C (PP2C) was another key regulator and got involved in inhibiting SnRK’s substrate phosphorylation which caused signaling cascade in the ABA signaling pathway^38^. PP2C-47 (gi|357147223) was found and down regulated at 6 h, which was in agreement with Sheard’s study^38^: PP2C acted as a constitutive negative regulaor of SnRKs whose autophosphorylation is required for kinase activity towards downstream targets. Thus, it indicated that Bd21 responded positively to defense the drought stress with phosphorylation at 6 h (Supplemental Table S2a)

In plants, the signals from the upstream elicitor receptors/sensors to the downstream mitogen-activated protein kinase (MAPK) substrates largely occur in three steps: the MAP kinase kinase kinase (MAPKKK), MAP kinase kinase (MAPKK), and MAPK cascade^39^. Some experiments have shown that various MAPK cascade members are involved in mediating signal transduction by transcription regulating P and N deprivation^40^. The activation of these MAP kinases is a sequential process through the phosphorylation of some conserved residues within an activation loop in their kinase domains^41^. In our study, three phosphorylated proteins were identified: MPK17 (gi|405778409), MPK7-1 (gi|405778403), and MPK3 (gi|405778399) (Table S1). MPK17 had two phosphorylation sites (Ser465, Ser491) and the phosphorylation level at Ser465 had significant up-regulation at 24 h. A recent study showed that MAP kinases could serve as upstream activators of the stress marker genes under saline conditions^41^. *A. thaliana* MPK20 and proline dehydrogenase might interact with each other and be part of a functional hypoosmotic stress-signaling pathway^42^. Sequence alignment and conservation analyses demonstrated that the expression of MPK17 was high, similar to *Arabidopsis thaliana* (Fig. 5; Table S5c). In addition, we identified some important CDPKs, such as CDPK26-like (gi|357125710; Table S2a) phosphorylated at Ser18, which supports the view that many of the autophosphorylation sites in CDPKs are localized at the N terminus.

### Transcription factors involved in gene expression

Under abiotic stress, many defense-related genes are regulated by transcription factors (TFs). In the present study, many TFs were phosphorylated (Table S2; Table 2). For example, nuclear TFs Y subunit A-7-like isoforms X1 (NF-Y A, gi|721672572), NF-Y B-1 (gi|357122032), and NF-Y beta (gi|721661240) were phosphorylated at Ser202 (_GAS(ph)PANQTGNRE_), Ser4 (_PDS(ph)DNEDS GGGGGIGGGGNNK_), and Ser105 (_LDTLDS(ph)PK_), respectively. Both NF-Y B and NF-Y A proteins were up-regulated, whereas the NF-Y beta protein was down-regulated. Some studies involving maize, *Arabidopsis*, and wheat have proven that NF-Y B subunits may respond to drought stress^43^,^44^. Nelson *et al.*^43^ reported tolerance to drought stress in transgenic maize, based on increasing ZmNFYB2 expression. The Bd21 phosphorylation levels were conserved (Fig. 5; Table S5c). Thus, these TFs responded positively to abiotic stresses.

Heat shock proteins (Hsps) and heat shock TFs (Hsfs) are involved in cellular responses to various sources of stress beyond heat stress^45^. The conformation of Hsps is affected by phosphorylation modification, such as the formation of active sites, flexibility, and inter-domain communication^46^. In other researches, HsfA1, as a main regulator, could enhance thermo tolerance and other stresses tolerance^47^,^48^. In our study, HsfA-2e-like (gi|357111341) was identified in Table S2a and HsfA-2e-like and Hsp81-1 (gi|357148345) have a complex relationship with other SCPL phosphoproteins (Figs 6 and S8). Hsfs recognized and combined with the Hsps gene promoter region of the heat shock element conservation motif. Thus, Hsfs may regulate Hsps transcription and gene expression to improve the stress tolerance of plants.

### Phosphoproteins involved in water and ion transport

In the face of various biotic and abiotic stresses, particularly drought stress, plants gradually adjust water and ion transport mechanisms. AQPs belong to a membrane protein family that rapidly transports water under osmotic pressure^49^. In our study, three AQPs were identified as belonging to the plasma membrane intrinsic protein (PIP) and nodulin 26-like intrinsic protein (NIP) subfamilies (Table S2a). All were up-regulated at 6 h under drought stress, which indicates that they had the same function in this process. NIP2-2 and PIP2-7 were phosphorylated at Ser271 and Ser282, respectively, in agreement with Guenther *et al.*^50^. Nodulin 26 phosphorylation at the C terminal tail (at Ser262) enhances water transport and is involved in the Ca^2+^ signal pathway to accelerate regulation of the water channel switch. Thus, phosphorylation could influence the activity of these AQPs.

There are three types of H^+^-ATPase: F-type, V-type, and P-type. The P-type is a large family of molecular pumps that act as intermediates in ATP hydrolysis and ion transport or ion counter-transport^51^,^52^. Moreover, activation of the plasma membrane H^+^-ATPase is associated with phosphorylation of a penultimate Thr and binding to the phosphorylated C-terminus of 14-3-3 protein to control the stomatal aperture^53^. A recent study has showed that the stomatal aperture is a limiting factor in photosynthesis and plant growth, and the overexpression of H^+^-ATPase in guard cells is useful for promoting plant growth^54^. We also identified some proteins related to H^+^-ATPase. Plasma membrane ATPase (gi|357166497) and V-type proton ATPase subunit a1 (gi|357125740) were up-regulated at 6 h and phosphorylated at Thr881 and Ser688 (Table S2a), which enhanced enzyme activity and related to osmotic regulation.

### ROS detoxification and stress defense

Many biotic and abiotic stresses might induce plant cells to produce ROS, leading to the acceleration of lipid peroxidation and leaf damage^55^,^56^ or beyond the antioxidant system^57^, particularly when subjected to drought stress. ROS scavenging or detoxification is a defense strategy in plants, involving a series of enzymes and proteins that reduce oxidative damage under drought stress^58^,^59^. In this work, one of the probable phospholipid hydroperoxide glutathione peroxidases (PHGPx, gi|357165189) was identified as an SCPL protein (Table S2b), which displayed up-regulated at 24 h. Phosphorylated PHGPx could become active to eliminate ROS and play a key role in the detoxification response to drought stress.

In addition, to sustain normal morphological structure and physiological function, plant cells must complete normal homeostasis, including electron transfer and oxidation reduction, and activate oxygen-scavenging to resist stress. In this study, we identified photosynthesis- and electron transport-related phosphorylated proteins that alleviate stress from drought. Chlorophyll a-b binding protein (chloroplast; LHCB, gi|357121229) was identified to be a specific SCPL protein at 6 h, and had significant up-regulation, as identified in salt stress (Table S2a; Table 2). LHCB was regulated by the redox state of the plastoquinone mediating electron transfer between photosystems I and II, which involve reversible phosphorylation. Moreover, LHCB occurred redox dependent phosphorylation by specific thylakoid-bound kinases to affect the reconstruction of light harvesting complexes^60^. Furthermore, the changes in physiological and ultrastructure (Fig. 1) also implied that chloroplast structure was injured under drought stress and LHCB phosphorylation activity was weakened, which influenced photosynthesis and energy metabolism.

LEA proteins are made up of a group of dehydration-inducible protein families and their function is to help maintain the minimum cellular water requirement. Comparison of dehydrin DHN3-like (gi|357149091, Table S1) with a LEA protein (RAB-17) in maize^61^, showed the same sequence motif of –SSSSSSSEDD– (Table S1). Two LEAs were identified in SCPL proteins (LEA1, gi|721622359; LWAB19.3, gi|721639026) and up-regulated at Ser81 and Ser91. In maize cells, phosphorylation of LEA could facilitate binding to the targeted proteins and involve nuclear location^62^. Moreover, LEA is involved in ABA induction under drought stress.

In addition, molecular chaperones were involved in regulating plant growth processes and the chaperone complexes of Hsp90 could respond to ABA stimulation activitily^63^. Hsp81-1 (gi|357148345), a molecular chaperone member of the Hsp90 family, has the same motif with some identified sequence phosphopeptides, such as -TTEKEIS(ph)DDEDEEEK- in *A. thaliana*^64^, rice^65^, and wheat^63^ (Table S5a). Hsp90 plays a key role in protein folding^66^ and participates in signal-transduction networks and protein degradation and trafficking^67^,^68^,^69^. Moreover, in the present study, Hsp81-1 was up-regulated at 6 h (Table S2a), suggesting that it could play an active role in response to drought stress.

### A putative pathway of drought stress responses in Bd21 leaves through phosphorylation modification

Based on the results from this study and previous studies, we propose a putative pathway of drought stress response in Bd21 seedling leaves through phosphorylation modification (Fig. 6). When plants were subjected to drought stress, membrane sensors sensed external environment changes, and transferred the chemical signals to the cell, which involved ABA, Ca^2+^, and MAPK signal pathways. Phosphate kinases/phosphatases were activated by the corresponding signal molecules. Then downstream TFs and translation factors were regulated by these phosphate kinases/phosphatases and they induced the production of drought stress response proteins such as LEA, Hsps, and LHCB. Massive accumulation of free radicals and ROS caused membrane lipid peroxidation, damaging the membrane systems, which caused the up-regulation of ROS scavenging-related phosphoproteins to defend against drought stress. At the same time, ABA and Ca^2+^ concentrations triggered stomata closure to defend against water stress. The AQPs and transporters on membranes also regulated the balance of osmosis by phosphorylation/dephosphorylation.

## Materials and Methods

### Plant materials and drought treatment

Bd21 kindly provided by Dr. John Vogel *et al.*^24^ at USDA-ARS, was used as plant material in this study. Bd21 seeds were surface sterilized by 15% sodium hypochlorite for 3 min, and rinsed 4 times in sterile distilled water. Seeds were submerged in water for 12 h at room temperature, and then transferred to wet filter paper to germinate at room temperature (22–25 °C) for 24 h. The uniformly germinated seeds were selected to grow in plastic pots containing Hoagland solution, in which was changed every two days. In a greenhouse, the experimental conditions included daily photo cycle of 16 h light/8 h dark (26 °C/18 °C) and 65–75% air humidity. Three biological replicates were performed under the same conditions. At the three leaf stage, the seedlings for different drought treatment times (0, 6, 12, 24 and 48 h) were cultivated in Hoagland solution containing 20% PEG6000^70^ (ψs = −0.75) measured by VAPRO pressure osmometer (Wescor 5520, USA), whereas the seedlings for the control were only cultivated in Hoagland solution (ψs = −0.045). After treatment for different times, some sampled leaves for each biological replicates were used for physiological indicator measurement and ultrastructure observation, and the remaining leaves were kept frozen in −80 °C for later protein extraction.

### Seedling morphology, physiological parameter measurement and leaf ultrastructure observation

Phenotypic, physiological characterization and ultrastructure of Bd21 seedling leaves under the control and treatment conditions were assessed. RWC measurements were conducted according to Larbi & Mekliche^71^ with minor modifications. Proline content and MDA content were measured, according to Bates *et al.*^72^ and Zhao *et al.*^73^ with minor modifications, respectively. Chlorophyll (chlorophyll a and b) were determined according to Zhang *at al.*^19^. Statistical analyses of the physiological measurements were conducted using an analysis of independent Student’s T-tests by SPSS statistics software (17.0). For ultrastructural observation by TEM, conventional chemical fixation, dehydration and embedding of 2 mm × 2 mm leaves segments were performed, according to the procedure described by Thiel *et al.*^74^. For electron microscopic analysis with a H-7500 transmission electron microscope (HITACHI Company, Japan).

### Protein extraction

Leaf total proteins were extracted according to the method of Lv *et al.*^21^ with minor modifications. Approximately 500 mg of fresh leaves from each biological replicate was ground into a fine powder in liquid nitrogen. The ground powder was suspended in 4 mL SDS buffer (30% sucrose, 2% SDS, 100 mM Tris-HCl, pH 8.0, 50 mM EDTA-Na_2_, 20 mM DTT) and 4 mL phenol (Tris-buffered, pH 8.0) in a 10 mL tube, followed by the addition of 1 mM phenylmethanesulfonyl fluoride (PMSF) and 20 μL protease inhibitor cocktail (Merck KGaA, Germany) per 1 g of fresh sample to inhibit the protease activities. Samples were mixed vigorously for 15 min at room temperature, centrifuged twice at 15700× g for 15 min each, and the supernatants were precipitated with 100 mM cold ethanolamine-methanol solution at −20 °C over night. After centrifuging at 15700× g for 15 min, the pellets were rinsed with cold acetone (−20 °C) and further centrifuged three times. After freeze-drying, the pellets were added to 300 μL of solubilization buffer (7 M urea, 2 M Thiourea, 1% DTT (w/v) and 4% CHAPS) at room temperature for 2 h. Insoluble materials were removed by centrifugation at 15700× g for 15 min, and protein samples were measured using a 2-D Quant Kit (Amersham Bioscience, USA). The final protein solution was stored at −80 °C for later use.

### Phosphopeptide enrichment using TiO_2_ microcolumns

Extracted protein mixtures were directly reduced with dithiothreitol (DTT), alkylated with iodoacetamide, and subsequently digested with endoproteinase Lys-C and trypsin as described previously^75^. The enrichment for phosphopeptides procedure was performed as reported by Wu *et al.*^76^ with some modifications. In detail, the TiO_2_ beads (GL Sciences, Tokyo, Japan) were incubated in 400 μL loading buffer containing 65% Acetonitrile (ACN)/2% trifluoroacetic acid (TFA)/saturated by glutamic acid. A total of 2 mg tryptic peptide were resolved in 600 μL loading buffer, and then incubated with appropriate amount of the TiO_2_ beads. After washing by 600 μL wash buffer (65% ACN/0.1% TFA), phosphopeptides were eluted twice with 300 μL elution buffer (500 mM NH4OH/60% ACN). The eluates were dried down and reconstituted in 0.1% formic acid (FA)/H_2_O for MS analysis.

### Phosphopeptide identification using LC-MS/MS

Enriched phosphopeptides were separated on a self-packed C18 reversed phase column (75 μm I. D., 150 mm length) (Column Technology Inc.), which directly connected on the nano electrospray ion source on a LTQ-Orbitrap XL mass spectrometer (Thermo Fisher Scientific). Pump flow was split to achieve a flow rate at 1 μL/min for sample loading and 300 nL/min for MS analysis. The mobile phases consisted of 0.1% FA (A) and 0.1% FA and 80% ACN (B). A five-step linear gradient of 5% to 30% B in 105 min, 35% to 90% B in 16 min, 90% B in 4 min, 90% to 2% B for 0.5 min and 2% B for 14.5 min was employed. The spray voltage was set to 2.0 kV and the temperature of heated capillary was 240 °C. For each sample, triplicate LC-MS/MS experiments were performed.

For data acquisition, each MS scan was acquired at a resolution of 60,000 (at 400 m/z) with lock mass option enabled and was followed by a data-dependent top 10 MS/MS scans using collision induced dissociation (CID). The threshold for precursor ion selection was 500. Mass window for precursor ion selection was 2.0 Da. The dynamic exclusion duration was 120 s, repeat count was 1 and repeat duration was 30 s. The analyzer for the MS scans was Orbitrap and for the MS/MS scans was LTQ (37% relative collision energy). Three biological replicates were performed from sample collection to the phosphopeptide identification using LC-MS/MS.

The raw files were processed with Maxquant (version 1.2.2.25)^77^ and searched against own *Brachypodium distachyon* L. Database (NCBI_B. distachyon_25824 entries_20150320. Fasta). Up to two missing cleavage points were allowed. The precursor ion mass tolerances were 7 ppm, and fragment ion mass tolerance was 0.5 Da for MS/MS spectra. The false discovery rate (FDR) was set to < 1.0% for both peptide and protein identification, the minimum peptide length was set to 6.

### Label free quantification and phosphorylation residue localization

Label free quantification was similar to the qualitative analysis on the experimental operation, but it needed more complex data requirements in the data extraction and processing methods. Each sample should be measured at least three times in parallel, in order to eliminate accidental errors. These datas of each phosphopeptide were normalized in calculated. MaxQuant software was used to calculated the integrating the ion intensities over its chromatographic elution profile coupled^26^,^77^,^78^. Phosphorylation residue localization was evaluated based on PTM scores that assign probabilities for each of the possible residues according to their residue-determining ions. In this study, Maxquant (version 1.2.2.25) was used to calculate PTM scores and PTM localization probabilities. Then, potential phosphorylation residues were grouped into three categories depending on their PTM localization probabilities^75^ namely class I (localization probability, *p* ≥ 0.9), class II (0.9 > *p* ≥ 0.75), class III (0.75 > *p* ≥ 0.5) and class IV (*p* < 0.5). The FDR of 1% was used for phosphorylation residues identification. Spectra without residue-determining ions resulted in the identification of phosphopeptides with undetermined residues.

### Verification of identified phosphoproteins by Pro-Q diamond gel staining

Extracted leaf total proteins were separated by 2-DE and then 2D gels were stained with Pro-Q Diamond (Invitrogen, USA) according to the manufacturer’s instructions. Briefly, the gels were fixed twice for 30 min/each time, and washed three times with ddH_2_O for 10 min/each time. The gels were incubated in Pro-Q Diamond stain in darkness for 2 h. The gels were destained with 20% acetonitrile in 50 mM sodium acetate (pH 4.0) for four times (30 min/each time), and then thrice washed for 5 min/each time. Finally, the gels were scanned on a TyphoonTM 9400 scanner (GE Healthcare, USA) with a 532 nm excitation laser and a 610 nm long pass filter with a resolution of 100 microns. After fluorescent image acquisition, the gels were stained with CBB to visualize total proteins. In addition, the same 2D gels were stained with CBB to visualize total proteins and used as control. The protein spots stained by Pro-Q Diamond were further identified by matrix-assisted laser desorption/ionization time-of-flight/time-of-flight mass spectrometry (MALDI-TOF/TOF MS) according to Lv *et al.*^21^.

### Bioinformatics

The significantly enriched phosphorylation motifs were extracted from the Motif-X algorithm^79^ (http://motif-x.med.harvard.edu/). Search Tool for the Retrieval of Interacting Genes/Proteins (STRING version 9.1) database of physical and functional interactions was used to analyze the Protein-Protein Interaction (PPI) of all the phosphoproteins identified in the current study^80^ (http://string-db.org). The sequences of all phosphoproteins were blasted in NCBI. Function annotations were based on Blast2Go^81^,^82^ (http://www.blast2go.com/b2ghome). Using EuKaryotic Orthologous Groups database (http://eggnog.embl.de/version_3.0/), KOG search and numbers were obtained. Then a dataset containing all the KOG numbers was used to do PPI analysis by using STRING and the interested network was displayed by Cytoscape (version 3.1.0) software (http://www.cytoscape.org/). Secondary structure of phosphoproteins was predicted via SABLE server version 2 (http://sable.cchmc.org/). Phyre2 online server (/redict the 3D structure of some interested phosphoproteins. Then the phosphorylated residues were displayed by SPDBV (version 4.1) software^83^ (http://spdbv.vital-it.ch/).

## Additional Information

**How to cite this article**: Yuan, L.-L. *et al.* Dynamic Phosphoproteome Analysis of Seedling Leaves in *Brachypodium distachyon* L. Reveals Central Phosphorylated Proteins Involved in the Drought Stress Response. *Sci. Rep.* **6**, 35280; doi: 10.1038/srep35280 (2016).

## Supplementary Material

Supplementary Information

Supplementary Tables

## Figures and Tables

**Table 1:** SCPL phosphoproteins of aquaporins under drought stress.

Proteins gi No.	Protein description	Number of Phospho (STY)	Amino acid	Modified sequences
gi|357124731	aquaporin NIP2-2	1	S	_FEEAPS(ph)VK_
gi|357119745	probable polyamine transporter At3g13620	1	S	_ETHLGDAS(ph)PKPK_
gi|357148345	heat shock protein 81–1	1	S	_EIS(ph)DDEDEEEKK_
gi|357125068	pyrophosphate-energized vacuolar membrane proton pump	1	S	_YIEAGNS(ph)EHAR_
gi|357142061	probable plastidic glucose transporter 2	1	S	_VSS(ph)RDAAMDPDVEMPVK_
gi|357137363	vacuolar amino acid transporter 1	1	S	_HLLGS(ph)VRDEK_
gi|357140450	monosaccharide-sensing protein 2-like	1	S	_DIAHHDHHGS(ph)TLGMR_
gi|357144310	choline transporter-like protein 2	1	S	_HAS(ph)AAAAGAGGEK_
gi|357134498	zinc transporter 6	1	S	_KAPTVFELAGEMS(ph)PR_
gi|721641127	metal tolerance protein 1-like isoform X1	1	S	_QHS(ph)HSSTGQHQGAEEPLLK_
gi|357168333	vacuolar cation/proton exchanger 1b-like	1	S	_S(ph)DASLLR_
gi|357147215	monosaccharide-sensing protein 2-like	1	S	_EIAAPHGS(ph)IMGAVGR_
gi|357167680	chloride channel protein CLC-c-like	1	S	_DGS(ph)FNYDIESM(ox)DGGGWR_
gi|357134243	ABC transporter G family member 12-like	1	S	_(ac)GEGNGVAWAGALS(ph)PAAR_
gi|357154538	cationic amino acid transporter 5-like	1	T	_APAET(ph)PPRDDHAPK_
gi|721615158	protein NRT1/PTR FAMILY 4.6 isoform X1	1	S	_APS(ph)PTGSTDMK_
gi|357113256	metal tolerance protein 4	1	S	_RNS(ph)VGSMR_
gi|357136945	mechanosensitive ion channel protein 6-like	1;2	S	_MAEKPGQSPS(ph)GR_
gi|357149489	ethylene-insensitive protein 2-like	1	T	_KTELSDT(ph)MK_
gi|357152022	heat shock protein 83	1	S	_AQS(ph)MGDTSSLDFMR_
gi|357159722	probable aquaporin PIP2-7	1;2	S	_S(ph)TGAATAR_
gi|357125740	V-type proton ATPase subunit a1	1	S	_FLGTSEM(ox)DPDSEPDS(ph)AR_
gi|357166028	ABC transporter C family member 2-like	1	S	_KEES(ph)KLQDIQR_
gi|357165526	calcium-transporting ATPase 8, plasma membrane-type	1	S	_EADDEKEGS(ph)AKQNNV_
gi|357137703	aquaporin PIP1-5	1	S	_LGANRYS(ph)ER_
gi|721610873	UDP-galactose transporter 2-like	1	S	_NKS(ph)DMLDGEDVPLK_

**Table 2:** Common SCPL phosphoproteins of different periods under drought stress compared with salt stress.

id	Proteins	protein name	Amino acid	Modified sequence
86	gi|357110863	LOW QUALITY PROTEIN: BUD13 homolog	S	_DSEEPQDLS(ph)PPR_
436	gi|357114107	uncharacterized protein LOC100836692	S	_PRPPAPALETIQS(ph)FAAR_
479	gi|357114452	paramyosin-like	S	_LES(ph)DLSASEASHVHEAELK_
500	gi|357115449	transcriptional adapter ADA2	S	_SNATVDSGGRDS(ph)PK_
648	gi|357118810	inositol hexakisphosphate and diphosphoinositol-pentakispho	S	_QGS(ph)GIIGTFGQSEELR_
794	gi|357121565	probable nucleolar protein 5–2	S	_DSEES(ph)PAADADGGEK_
801	gi|357121608	nucleolar protein 56-like	S	_NRDVS(ph)EDAEPK_
802	gi|357121619	protein EARLY RESPONSIVE TO DEHYDRATION	S	_AQLSVVNS(ph)PR_
838	gi|357122032	nuclear transcription factor Y subunit B-1-like	S	_PDS(ph)DNEDSGGGGGIGGGGNNK_
955	gi|357122930	calreticulin-like	S	_DDS(ph)DDEKPQHANKDEK_
982	gi|357123075	epidermal growth factor receptor substrate 15-like 1	S	_DNNLFGQRDS(ph)FSR_
987	gi|357123075	epidermal growth factor receptor substrate 15-like 1	S	_GASDS(ph)PVHGDKANDR_
1106	gi|357124170	uncharacterized protein LOC100825390	S	_LETILS(ph)GK_
5715	gi|357126063	photosystem II 22 kDa protein, chloroplastic	T	_PKT(ph)EDGIFGTSGGIGFTK_
1330	gi|357126396	vacuolar protein sorting-associated protein 53 A	S	_NKELES(ph)DDENEGVEQNK_
1607	gi|357132564	splicing factor U2af small subunit B	S	_GRS(ph)PVRENS(ph)EER_
1821	gi|357134498	zinc transporter 6	S	_KAPTVFELAGEMS(ph)PR_
1847	gi|357134721	uncharacterized protein LOC100835223 I	S	_ATS(ph)PPPAAENLAVSVVR_
1891	gi|357135111	uncharacterized protein LOC100843157 isoform X1	S	_RAVEES(ph)DEEPEEK_
1893	gi|357135111	uncharacterized protein LOC100843157 isoform X1	S	_FTQQSTS(ph)DDDDDKAAAEPPK_
1927	gi|357135514	uncharacterized protein At1g03900	S	_LPPPPPS(ph)PVSPTDSGVPFSPFK_
5833	gi|357136108	uncharacterized protein LOC100838789	T	_GST(ph)PTSAPAHVHPK_
2004	gi|357136280	GTP-binding protein At2g22870	S	_TVDHS(ph)DAEGSR_
2008	gi|357136419	serine/arginine repetitive matrix protein 2-like	S	_GFFFHPS(ph)PR_
2056	gi|357136945	mechanosensitive ion channel protein 6-like I	S	_MAEKPGQSPS(ph)GR_
2123	gi|357137491	clathrininteractor EPSIN 2-like	S	_GSNSNPEYAEGS(ph)GRR_
2286	gi|357140450	monosaccharide-sensing protein 2-like	S	_DIAHHDHHGS(ph)TLGMR_
2289	gi|357140450	monosaccharide-sensing protein 2-like	S	_IYLHQEGVPDS(ph)R_
2499	gi|357144952	RNA polymerase II-associated factor 1 homolog	S	_QRS(ph)SLDDDLDEHPK_
2505	gi|357145005	DNA topoisomerase 1-like	S	_KADDS(ph)DDDHKPLSLK_
2542	gi|357145548	probable sucrose-phosphate synthase 4	S	_IGS(ph)TDAIEVWANQHK_
2683	gi|357147169	U2 snRNP-associated SURP motif-containing protein	S	_WNRDDDVS(ph)DDENRK_
2692	gi|357147215	monosaccharide-sensing protein 2-like	S	_EIAAPHGS(ph)IMGAVGR_
2696	gi|357147215	monosaccharide-sensing protein 2-like	S	_HGS(ph)MVSQGK_
5973	gi|357148313	pre-mRNA-splicing factor CWC25 homolog	T	_RQQNT(ph)PEDGEPR_
2783	gi|357148363	mediator of RNA polymerase II transcription subunit 15	S	_GAVVAES(ph)PR_
2801	gi|357148434	putative DNA-binding protein ESCAROLA	S	_GTLSESSGGTAS(ph)PR_
2899	gi|357149553	chloride channel protein CLC-c-like	S	_EGS(ph)HNLDIESM(ox)DGGGGGDWR_
6005	gi|357150108	muscle M-line assembly protein unc-89	T	_DKPCNT(ph)EDAEDVGQLK_
2991	gi|357151448	RNA polymerase-associated protein RTF1 homolog	S	_EDEFDES(ph)PSR_
3060	gi|357154143	splicing factor U2af small subunit A-like	S	_GGDYYGGS(ph)LDR_
3093	gi|357154529	uncharacterized protein LOC100830146	S	_TGS(ph)SSSSLFAR_
3203	gi|357157075	probable sucrose-phosphate synthase 5	S	_EAAEELS(ph)EGEKEK_
3206	gi|357157075	probable sucrose-phosphate synthase 5	S	_LEPAPALGLAAEESGAGAGAAYS(ph)PTR_
3235	gi|357157399	fructose-bisphosphatealdolase,chloroplastic	S	_TANTIAS(ph)PGR_
3244	gi|357157443	sec1 family domain-containing protein MIP3	S	_KKDLS(ph)DDELGQVEAR_
3408	gi|357160417	serine/arginine-rich SC35-like splicing factor SCL30	S	_GYGGS(ph)PPHR_
3544	gi|357163483	uncharacterized protein LOC100825367	S	_GDIAEQGS(ph)FHAEDDR_
3600	gi|357164525	uncharacterized protein LOC100825062 isoform X1	S	_EKEVSS(ph)EDEEQGSAK_
3603	gi|357164525	uncharacterized protein LOC100825062 isoform X1	S	_VS(ph)NKDEAVSTK_
3830	gi|357168405	zinc finger CCCH domain-containing protein 13	S	_YDPNSNELS(ph)DDENRDR_

## References

[b1] Barnabás B., Jäger K. & Fehér A. The effect of drought and heat stress on reproductive processes in cereals. Plant Cell Environ. 31, 11–38 (2008). 17971069 10.1111/j.1365-3040.2007.01727.x

[b2] Harb A., Krishnan A., Ambavaram M. M. & Pereira A. Molecular and physiological analysis of drought stress in Arabidopsis reveals early responses leading to acclimation in plant growth. Plant Physiol. 154, 1254–1271(2010). 20807999 10.1104/pp.110.161752 PMC2971604

[b3] Zhu J. K. Salt and drought stress signal transduction in plants. Ann Rev Plant Biol. 53, 247 (2002). 12221975 10.1146/annurev.arplant.53.091401.143329 PMC3128348

[b4] Wilkinson S. & Davies W. J., Drought, ozone, ABA and ethylene: new insights from cell to plant to community. Plant, Cell Environ. 33, 510–525 (2010). 19843256 10.1111/j.1365-3040.2009.02052.x

[b5] Lee S. C. & Luan S. ABA signal transduction at the crossroad of biotic and abiotic stress responses. Plant, Cell Environ. 35, 53–60 (2012). 21923759 10.1111/j.1365-3040.2011.02426.x

[b6] Berridge M. J., Lipp P. & Bootman M. D. The versatility and universality of calcium signaling. Nat Rev Mol Cell Biol. 1, 11–21 (2000). 11413485 10.1038/35036035

[b7] D’Autréaux B. & Toledano M. B. ROS as signalling molecules: mechanisms that generate specificity in ROS homeostasis. Nat Rev Mol Cell Biol. 8, 813–824 (2007). 17848967 10.1038/nrm2256

[b8] Finkel T. Signal transduction by reactive oxygen species. J Cell Biol. 194, 7–15 (2011). 21746850 10.1083/jcb.201102095 PMC3135394

[b9] Thingholm T. E., Jensen O. N. & Larsen M. R. Analytical strategies for phosphoproteomics. Proteomics. 9, 1451–1468 (2009). 19235172 10.1002/pmic.200800454

[b10] Melo-Braga M. N. *et al.* Modulation of protein phosphorylation, N-glycosylation and Lys-acetylation in grape (Vitisvinifera) mesocarp and exocarp owing to lobesiabotrana infection. Mol Cell Proteomics. 1, 945–956 (2012). 10.1074/mcp.M112.020214 PMC3494143 22778145

[b11] Tanou G. *et al.* Oxidative and nitrosative‐based signaling and associated post‐translational modifications orchestrate the acclimation of citrus plants to salinity stress. Plant J. 72, 585–599 (2012). 22780834 10.1111/j.1365-313X.2012.05100.x

[b12] Guo Y. *et al.* Casein kinase1-like protein2 regulates actin filament stability and stomatal closure via phosphorylation of actin depolymerizing factor. Plant Cell. 28, 1422–1439 (2016). 27268429 10.1105/tpc.16.00078 PMC4944410

[b13] Silva-Sanchez C., Li H. & Chen S. Recent advances and challenges in plant phosphoproteomics. Proteomics. 15, 1127–1141 (2015). 25429768 10.1002/pmic.201400410

[b14] Khan M., Takasaki H. & Komatsu S. Comprehensive phosphoproteome analysis in rice and identification of phosphoproteins responsive to different hormones/stresses. J Proteome Res. 4, 1592–1599 (2005). 16212411 10.1021/pr0501160

[b15] Kline K. G., Barrett-Wilt G. A. & Sussman M. R. In planta changes in protein phosphorylation induced by the plant hormone abscisic acid. Proc Natl Acad Sci. USA 107, 15986–15991 (2010). 20733066 10.1073/pnas.1007879107 PMC2936636

[b16] Nguyen T. H. N. *et al.* Quantitative phosphoproteomic analysis of soybean root hairs inoculated with *Bradyrhizobium japonicum*. Mol Cell Proteomic. 11, 1140–1155 (2012). 10.1074/mcp.M112.018028 PMC3494206 22843990

[b17] Bonhomme L., Valot B., Tardieu F. & Zivy M. Phosphoproteome dynamics upon changes in plant water status reveal early events associated with rapid growth adjustment in maize leaves. Mol Cell Proteomics. 11, 957–972 (2012). 22787273 10.1074/mcp.M111.015867 PMC3494150

[b18] Zhang M. *et al.* Comparative phosphoproteome analysis of the developing grains in bread wheat (*Triticum aestivum* L.) under well-watered and water-deficit conditions. J Proteome Res. 13, 4281–4297 (2014). 25145454 10.1021/pr500400t

[b19] Zhang M. *et al.* Phosphoproteome analysis reveals new drought response and defense mechanisms of seedling leaves in bread wheat (*Triticum aestivum* L.). J Proteomics. 109, 290–308 (2014). 25065648 10.1016/j.jprot.2014.07.010

[b20] Lv D. W. *et al.* Proteomic and phosphoproteomic analysis reveals the response and defense mechanism in leaves of diploid wheat *T. monococcum* under salt stress and recovery. J Proteomics. 143, 93–105 (2016). 27095598 10.1016/j.jprot.2016.04.013

[b21] Lv D. W. *et al.* Large-scale phosphoproteome analysis in seedling leaves of *Brachypodium distachyon* L. BMC Genomics. 15, 1 (2014). 24885693 10.1186/1471-2164-15-375 PMC4079959

[b22] Draper J. *et al.* *Brachypodium distachyon*. A new model system for functional genomics in grasses. Plant Physiol. 127, 1539–1555 (2001). 11743099 PMC133562

[b23] Douché T. *Brachypodium distachyon* as a model plant toward improved biofuel crops: Search for secreted proteins involved in biogenesis and disassembly of cell wall polymers. Proteomics. 13, 2438–2454 (2013). 23784962 10.1002/pmic.201200507

[b24] Vogel J. P. *et al.* Genome sequencing and analysis of the model grass *Brachypodium distachyon*. Nature. 463, 763–768 (2010). 20148030 10.1038/nature08747

[b25] Kosova K. *et al.* Proteome analysis of cold response in spring and winter wheat (*Triticum aestivum*) crowns reveals similarities in stress adaptation and differences in regulatory processes between the growth habits. J Proteome Res. 12, 4830–4845 (2013). 24047233 10.1021/pr400600g

[b26] Lv D. W. *et al.* Proteome and phosphoproteome characterization reveals new response and defense mechanisms of *Brachypodium distachyon* leaves under salt stress. Mol Cell Proteomics. 13, 632–652 (2014). 24335353 10.1074/mcp.M113.030171 PMC3916659

[b27] Sharma P. *et al.* Phosphorylation of mek1 by cdk5/p35 down-regulates the mitogen-activated protein kinase pathway. J Biol Chem. 277 **(1)**, 528–534 (2002) 11684694 10.1074/jbc.M109324200

[b28] Oesch-Bartlomowicz B. & Oesch F. Cytochrome-p450 phosphorylation as a functional switch. Arch Biochem Biophy. 409 **(1)**, 228–234 (2003). 10.1016/s0003-9861(02)00558-1 12464263

[b29] Lv D. W. *et al.* Integrative network analysis of the signaling cascades in seedling leaves of bread wheat by large-scale phosphoproteomic profiling. J Proteome Res. 13 **(5)**, 2381–2395 (2014). 24679076 10.1021/pr401184v

[b30] Yeong S. S. *et al.* The last 10 amino acid residues beyond the hydrophobic motif are critical for the catalytic competence and function of protein kinase Cα. J Biol Chem. 281 **(41)**, 30768–30781 (2006). 16895917 10.1074/jbc.M511278200

[b31] Yao Q. *et al.* P3DB: An integrated database for plant protein phosphorylation. Front Plant Sci. 3, 206 (2012). 22973285 10.3389/fpls.2012.00206 PMC3435559

[b32] Rose C. M. *et al.* Rapid phosphoproteomic and transcriptomics changes in the rhizobia-legume symbiosis. Mol Cell Proteomics. 11 **(9)**, 724−744 (2012). 22683509 10.1074/mcp.M112.019208 PMC3434772

[b33] Heazlewood J. L. *et al.* PhosPhAt: a database of phosphorylation sites in *Arabidopsis thaliana* and a plant-specific phosphorylation site predictor. Nucleic Acids Res. 36 **(D)**, D1015−D1021 (2008). 17984086 10.1093/nar/gkm812 PMC2238998

[b34] Guo M. & Huang B. X. Integration of phosphoproteomic, chemical, and biological strategies for the functional analysis of targeted protein phosphorylation. Proteomics. 13, 424–437 (2013). 23125184 10.1002/pmic.201200274

[b35] Park S. Y. *et al.* Abscisic acid inhibits type 2C protein phosphatases via the PYR/PYL family of START proteins. Science. 324, 1068–1071 (2009). 19407142 10.1126/science.1173041 PMC2827199

[b36] Gómez-Cadenas A. *et al.* An abscisic acid-induced protein kinase, PKABA1, mediates abscisic acid-suppressed gene expression in barley aleurone layers. Proc Natl Acad Sci. USA. 96, 1767–1772 (1999). 9990099 10.1073/pnas.96.4.1767 PMC15589

[b37] Zhang H., Mao X., Wang C. & Jing R. Overexpression of a common wheat gene *TaSnRK2. 8*enhances tolerance to drought, salt and low temperature in Arabidopsis. PLoS One. 5, e16041 (2010). 21209856 10.1371/journal.pone.0016041 PMC3012728

[b38] Sheard L. B. & Zheng N. Plant biology: signal advance for abscisic acid. Nature. 462 **(7273)**, 575–576 (2009). 19956245 10.1038/462575a

[b39] Meng X. & Zhang S. MAPK cascades in plant disease resistance signaling. Ann Rev Phytopath. 51, 245–266 (2013). 10.1146/annurev-phyto-082712-102314 23663002

[b40] Wen Y. *et al.* Characterization and expression analysis of mitogen-activated protein kinase cascade genes in wheat subjected to phosphorus and nitrogen deprivation, high salinity, and drought. J Plant Biochem Biotechnol. 24, 184–196 (2015).

[b41] Moustafa K., AbuQamar S., Jarrar M., Al-Rajab A. J. & Trémouillaux-Guiller J. MAPK cascades and major abiotic stresses. Plant Cell Rep. 33, 1217–1225 (2014). 24832772 10.1007/s00299-014-1629-0

[b42] Moustafa K., De Vos D. L., Leprince A. S., Savouré A. & Lauriere C. Analysis of the Arabidopsis mitogen-activated protein kinase families: organ specificity and transcriptional regulation upon water stresses. Sch Res Exch. 225, 196–214 (2008).

[b43] Nelson D. E. *et al.* Plant nuclear factor Y (NF-Y) B subunits confer drought tolerance and lead to improved corn yields on water-limited acres. Proc Natl Acad Sci. USA 104, 16450–16455 (2007). 17923671 10.1073/pnas.0707193104 PMC2034233

[b44] Petroni K. *et al.* The promiscuous life of plant NUCLEAR FACTOR Y transcription factors. Plant Cell. 24, 4777–4792 (2012). 23275578 10.1105/tpc.112.105734 PMC3556957

[b45] Swindell W. R., Huebner M. & Weber A. P. Transcriptional profiling of *Arabidopsis* heat shock proteins and transcription factors reveals extensive overlap between heat and non-heat stress response pathways. BMC Genomics. 8, 1 (2007). 17519032 10.1186/1471-2164-8-125 PMC1887538

[b46] Li J. & Buchner J. Structure, function and regulation of the hsp90 machinery. Biomed J 36, 106 (2013). 23806880 10.4103/2319-4170.113230

[b47] Mishra S. K., Tripp J., Winkelhaus S., Tschiersch B. & Theres K. In the complex family of heat stress transcription factors, HSFA1 has a unique role as master regulator of thermo tolerance in tomato. Gene Dev. 16, 1555–1567 (2002) 12080093 10.1101/gad.228802 PMC186353

[b48] Liu H. C., Liao H. T. & Charng Y. Y. The role of class A1 heat shock factors (HSFA1s) in response to heat and other stresses in *Arabidopsis*. Plant Cell Environ. 34. 738–751 (2011) 21241330 10.1111/j.1365-3040.2011.02278.x

[b49] Gomes D. *et al.* Aquaporins are multifunctional water and solute transporters highly divergent in living organisms. BBA-Biomembranes. 1788, 1213–1228 (2009). 19327343 10.1016/j.bbamem.2009.03.009

[b50] Guenther J. F. *et al.* Phosphorylation of soybean nodulin 26 on serine 262 enhances water permeability and is regulated developmentally and by osmotic signals. Plant Cell. 15, 981–991 (2003). 12671092 10.1105/tpc.009787 PMC152343

[b51] Morth J. P. *et al.* A structural overview of the plasma membrane Na^+^, K^+^-ATPase and H^+^-ATPase ion pumps. Nat Rev Mol Cell Bio. 12, 60–70 (2011). 21179061 10.1038/nrm3031

[b52] Wang H., Wu K., Liu Y., Wu Y. & Wang X. Integrative proteomics to understand the transmission mechanism of *Barley* yellow dwarf virus-GPV by its insect vector *Rhopalosiphum padi*. Sci Rep. 5. 10971 (2015) 26161807 10.1038/srep10971 PMC4498328

[b53] Hayashi M., Inoue S. I., Takahashi K. & Kinoshita T. Immunohistochemical detection of blue light-induced phosphorylation of the plasma membrane H^+^-ATPase in stomatal guard cells. Plant Cell Physiol. 52, 1238–1248 (2011). 21666226 10.1093/pcp/pcr072

[b54] Wang Y. *et al.* Overexpression of plasma membrane H^+^-ATPase in guard cells promotes light-induced stomatal opening and enhances plant growth. Proc Natl Acad Sci. USA 111, 533–538(2014). 24367097 10.1073/pnas.1305438111 PMC3890815

[b55] Upadhyaya H., Khan M. H. & Panda S. K. Hydrogen peroxide induces oxidative stress in detached leaves of *Oryza sativa* L. Plant Physiol. 33, 83–95 (2007).

[b56] Liu Y. & He C. Regulation of plant reactive oxygen species (ROS) in stress responses: learning from atrbohd. Plant Cell Rep. 35 **(5)**, 1–13 (2016). 26883222 10.1007/s00299-016-1950-x

[b57] Liu B., Qin F., Liu W. & Wang X. Differential proteomics profiling of the ova between healthy and rice stripe virus-infected female insects of *Laodelphax striatellus*. Sci Rep. 6, 27216 (2016). 27277140 10.1038/srep27216 PMC4899684

[b58] Asada K. Production and scavenging of reactive oxygen species in chloroplasts and their functions. Plant Physiol. 141, 391–396 (2006). 16760493 10.1104/pp.106.082040 PMC1475469

[b59] Bailey-Serres J. & Mittler R. The roles of reactive oxygen species in plant cells. Plant Physiol. 141, 311–311 (2006). 16760480 10.1104/pp.104.900191 PMC1475481

[b60] Kargul J. & Barber J. Photosynthetic acclimation: Structural reorganisation of light harvesting antenna–role of redox‐dependent phosphorylation of major and minor chlorophyll a/b binding proteins. FRBS J. 275, 1056–1068 (2008). 10.1111/j.1742-4658.2008.06262.x 18318833

[b61] Vilardell J. *et al.* Gene sequence, developmental expression, and protein phosphorylation ofRAB-17 in maize. Plant Mol Biol. 14, 423–432 (1990). 2151715 10.1007/BF00028778

[b62] Jensen A. B., Goday A., Figueras M. & Jessop A. C. Phosphorylation mediates the nuclear targeting of the maize Rab17 protein. Plant J. 13, 691–697 (1998). 9681011 10.1046/j.1365-313x.1998.00069.x

[b63] Hao P. C. *et al.* An integrative proteome analysis of different seedling organs in tolerant and sensitive wheat cultivars under drought stress and recovery. Proteomics. 15, 1544–1563 (2015). 25546360 10.1002/pmic.201400179

[b64] Ito J. *et al.* A survey of the *Arabidopsis thaliana* mitochondrial phosphoproteome. Proteomics. 9, 4229–4240 (2009). 19688752 10.1002/pmic.200900064

[b65] Nakagami H. *et al.* Large-scale comparative phosphoproteomics identifies conserved phosphorylation sites in plants. Plant Physiol. 153, 1161–1174 (2010). 20466843 10.1104/pp.110.157347 PMC2899915

[b66] Frydman J. Folding of newly translated proteins *in vivo*: the role of molecular chaperones. Ann Rev Biochem. 70, 603–647 (2001). 11395418 10.1146/annurev.biochem.70.1.603

[b67] Richter K. & Buchner J. Hsp90: chaperoning signal transduction. J Cell Physiol. 188, 281–290 (2001). 11473354 10.1002/jcp.1131

[b68] Pratt W. B., Krishna P. & Olsen L. J. Hsp90-binding immunophilins in plants: the protein movers. Trends Plant Sci. 6, 54–58 (2001). 11173288 10.1016/s1360-1385(00)01843-4

[b69] Young J. C., Moarefi I. & Hartl F. U. Hsp90: a specialized but essential proteinfolding tool. J Cell Biol. 154, 267–273 (2001). 11470816 10.1083/jcb.200104079 PMC2150759

[b70] Li Z. *et al.* The physiological and itraq-based proteomic analyses reveal the function of spermidine on improving drought tolerance in white clover. J Proteome Res. 15, 1563−1579 (2016). 27030016 10.1021/acs.jproteome.6b00027

[b71] Larbi A. & Mekliche A. Relative water content (RWC) and leaf senescence as screening tools for drought tolerance in wheat. Options Mediterr. Série ASém. Méditerr. 60, 193–196 (2004).

[b72] Bates L. S., Waldren R. P. & Teare I. D. Rapid determination of free proline for water-stress studies. Plant Soil. 39, 205–207 (1973).

[b73] Zhao S., Xu C. C., Zou Q. & Meng Q. W. Improvements of method for measurement of malondialdehyde in plant tissues. Physiol. Commun. 30, 207–210 (1994).

[b74] Thiel J. *et al.* Differentiation of endosperm transfer cells of barley: a comprehensive analysis at the micro-scale. Plant J. 71, 639–655 (2012). 22487146 10.1111/j.1365-313X.2012.05018.x

[b75] Olsen J. V. *et al.* Global, *in vivo*, and site-specific phosphorylation dynamics in signaling networks. Cell. 127, 635–648 (2006). 17081983 10.1016/j.cell.2006.09.026

[b76] Wu J. *et al.* Global profiling of phosphopeptides by titania affinity enrichment. J Proteome Res. 6, 4684–4689 (2007). 17929885 10.1021/pr070481m

[b77] Cox J. & Mann M. MaxQuant enables high peptide identification rates, individualized ppb-range mass accuracies and proteome-wide protein quantification. Nat Biotechnol. 26, 1367–1372 (2008). 19029910 10.1038/nbt.1511

[b78] Sandin M., Krogh M., Hansson K. & Levander F. Generic workflow for quality assessment of quantitative label-free LC–MS analysis. Proteomics. 11, 1114–1124 (2011). 21298787 10.1002/pmic.201000493

[b79] Schwartz D. & Gygi S. P. An iterative statistical approach to the identification of protein phosphorylation motifs from large-scale data sets. Nat Biotechnol. 23, 1391–1398 (2005). 16273072 10.1038/nbt1146

[b80] Franceschini A. *et al.* STRING v9. 1: protein-protein interaction networks, with increased coverage and integration. Nucleic Acids Res. 41, D808–D815 (2013). 23203871 10.1093/nar/gks1094 PMC3531103

[b81] Conesa A. & Götz S. Blast2GO: A comprehensive suite for functional analysis in plant genomics. Intl J Plant Genomics. 2008, 619832 (2008). 10.1155/2008/619832 PMC2375974 18483572

[b82] Liu W., Gray S., Huo Y., Li L., Wei T. & Wang X. Proteomic analysis of interaction between a plant virus and its vector insect reveals new functions of hemipteran cuticular protein. Mol Cell Proteomics. 14 **(8)**, 2229–2242 (2015). 26091699 10.1074/mcp.M114.046763 PMC4528249

[b83] Guex N. & Peitsch M. C. SWISS‐MODEL and the Swiss‐Pdb Viewer: an environment for comparative protein modeling. Electrophoresis. 18, 2714–2723 (1997). 9504803 10.1002/elps.1150181505

