# Supplementary material for: Dynamic Phosphoproteome Analysis of Seedling Leaves in Brachypodium distachyon L. Reveals Central Phosphorylated Proteins Involved in the Drought Stress Response
Source: Sci Rep. 2016 Oct 17;6:35280. doi: 10.1038/srep35280 (PMC5066223; doi:10.1038/srep35280)
Supplement: Supplementary Information [file srep35280-s1.doc]

**Dynamic** **Phosphoproteome Analysis of Seedling Leaves in** ***Brachypodium distachyon* L*.* Reveals Central Phosphorylated Proteins Involved in the Drought Stress Response**

**Lin-Lin** **Yuan****^1,3^, Ming Zhang^1,2,3^, Xing Yan^1,3^, Yan-Wei Bian^1^,**

**Shou-Min Zhen^1^, and Yue-Ming Yan^1*^**

**Supplemental Figure Legends:**

**Supplemental Figure S1. Phenotypic and physiological changes in *Bd21* leaves under drought stress.** A. Phenotypic appearance at 0, 6, 12, 24 and 48h. B. a. Relative water content (RWC) changes. b. Chlorophyll content changes. c. Malonaldehyde (MDA) content changes. d. Proline content changes. Error bars indicate standard errors of three biological replicates. Statistically significant differences compared to the control were calculated based on an independent Student's t-tests: **P* < 0.05; ***P* < 0.001.

**Supplemental Figure S2. Workflow of quantitative phosphoproteome analysis in *Bd21* seedling leaves under drought stress.** C: control condition; T: treatment conditions; C1～3, T6-1～3 and T24-1～3 represent three biological replicates under control, drought stress at 6 h and 24 h, respectively.

**Supplemental Figure S3. Phophorylation status of *Bd21* seedling leaves under drought stress.** a. The number of phosphosites, phosphopeptides and phosphoproteins. b. The number of phosphosites of each phosphopeptides. c. The counts of serine, threonine, and tyrosine residues of the total phosphorylation residues. d. Localization of phosphosites’s probability.

**Supplemental Figure S4. Phosphoproteins of Bd21 seedling leaves under drought stress at 6 h (a) and 24 h (b) by Pro-Q diamond gel staining.**

**Supplemental Figure S5. Protein expression cluster analysis of SCPL proteins.** Each row displays the change of a SCPL protein and each line represents all of the SCPL proteins at 0, 6 and 24 h.

**Supplemental Figure S6. Sequence alignment and three-dimensional structures for three AQPs in Bd21 seedling leaves.** a. Protein sequences compared with PIP1-5, IPIP2-7 and NIP2-2. The sequences with red box represent the phosphorylation sites. b. Three-dimensional structures of three AQPs.

**Supplemental Figure S7. Functional classification of SCPL proteins.** a. The functional classification of SCPL proteins in hierarchical clusters patterns I. b. The functional classification of SCPL proteins in hierarchical clusters patterns II. c. The functional classification of common SCPL phosphoproteins of different periods under drought stress compared with salt stress.

**Supplemental Figure S8. PPI networks of some SCPL phosphoproteins under drought stress in seedling leaves of Bd21.** Nodes with different background color represent the different functional categories.

**Supplemental Table Legends:**

**Supplemental Table S1. Total phosphoproteins were identified of Bd21 seeding leaves from this study.**

**Supplemental Table S2A. SCPL phosphoproteins at 0-6h in Bd21 seeding leaves under drought stress.**

**Supplemental Table S2B. SCPL phosphoproteins at 6-24h in Bd21 seeding leaves under drought stress.**

**Supplemental Table S3. The SCPL proteins expression clustering analysis of Bd21 leaf under drought stress treatment .**

**Supplemental Table S4. The GO annotation of SCPL phosphoproteins in Bd21 leaf under drought stress.**

**Supplemental Table S5A. The motif enrichment of SCPL phosphopeptides from Bd21 under drought stress.**

**Supplemental Table S5B. The secondary structure of SCPL phosphoproteins from Bd21 under drought stress.**

**Supplemental Table S5C: Conservation analysis of SCPL phosphoproteins identified from Bd21 under drought stress.**

**Supplemental Table S6: The KOG annotation of SCPL phosphoproteins from Bd21 under drought stress.**

**Supplemental Table S7. Verification of identified phosphoproteins by Pro-Q diamond gel stain by MALDI-TOF/TOF-MS from leaf samples.**


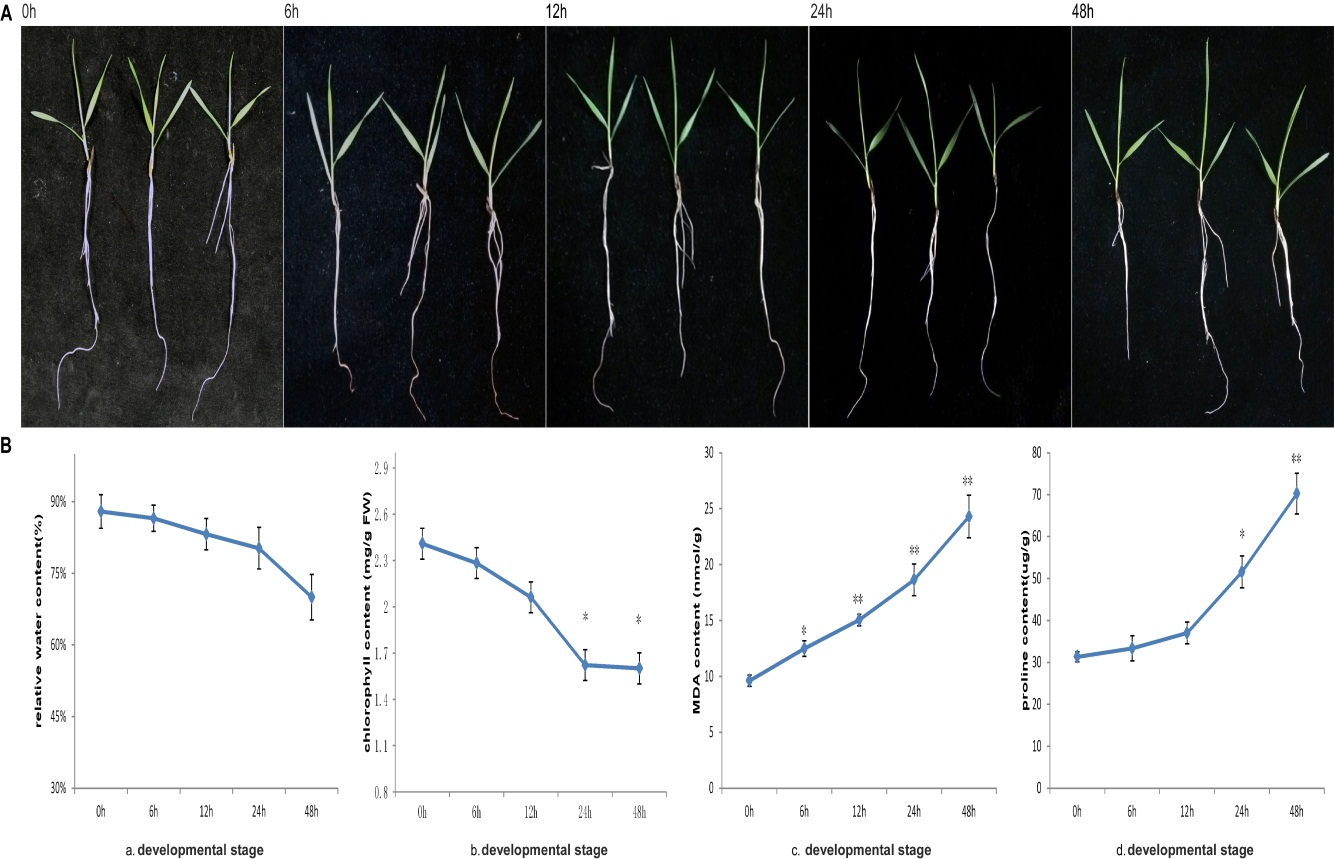


**Supplemental Figure S1**


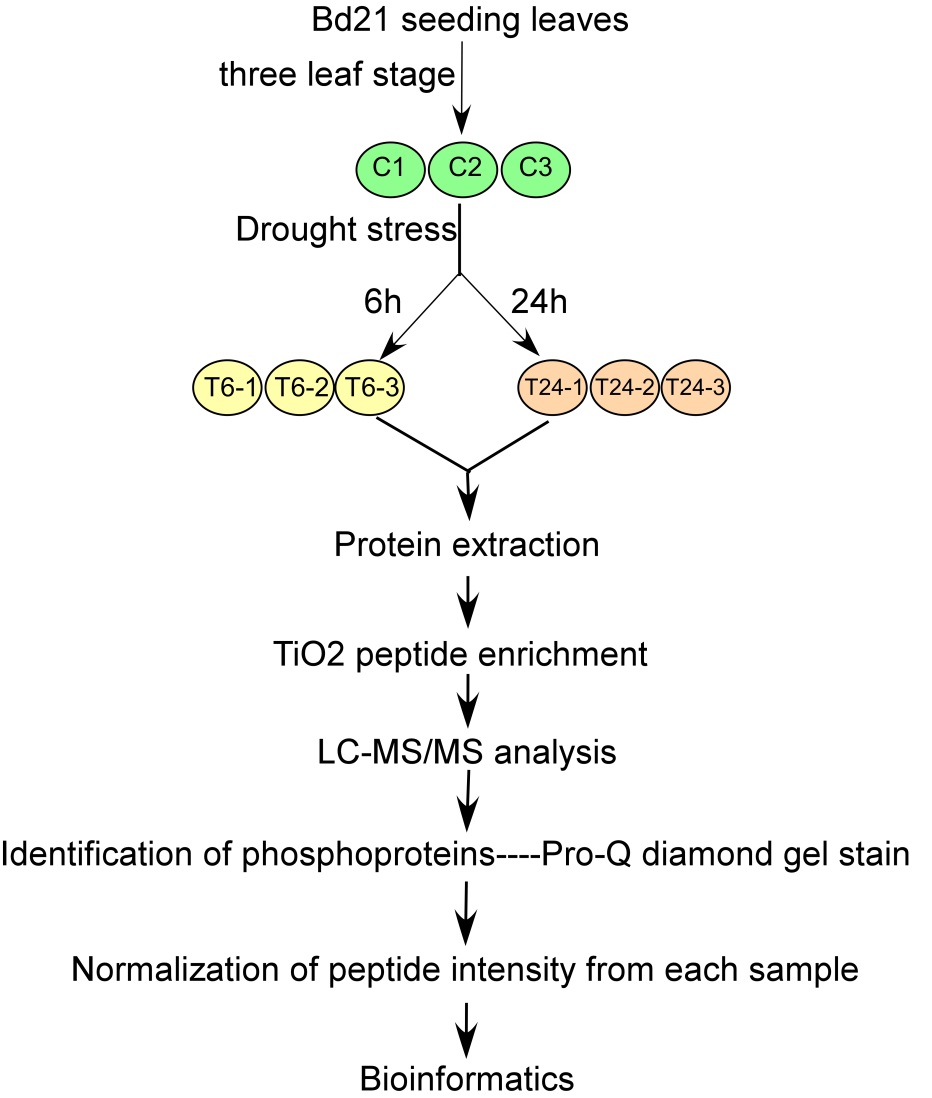


**Supplemental Figure S2**


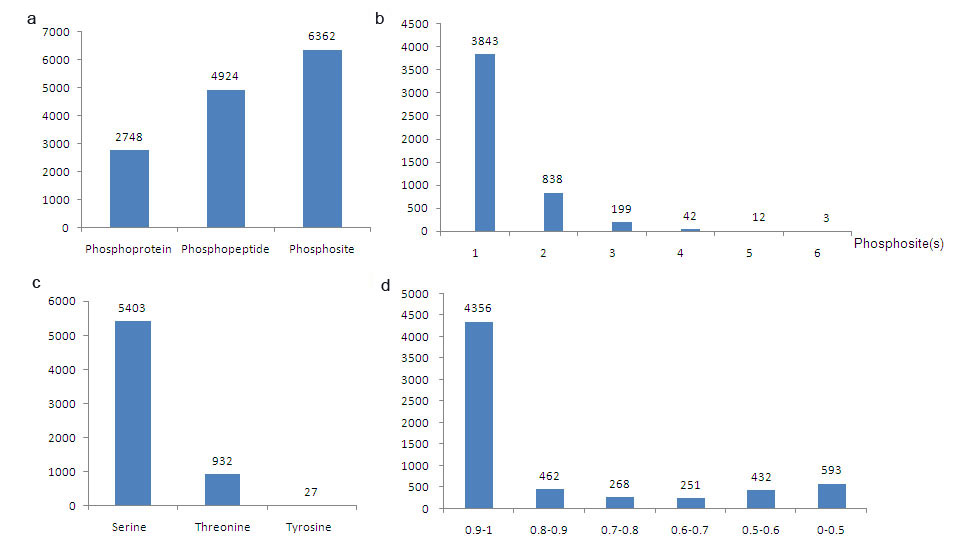


**Supplemental Figure S3**


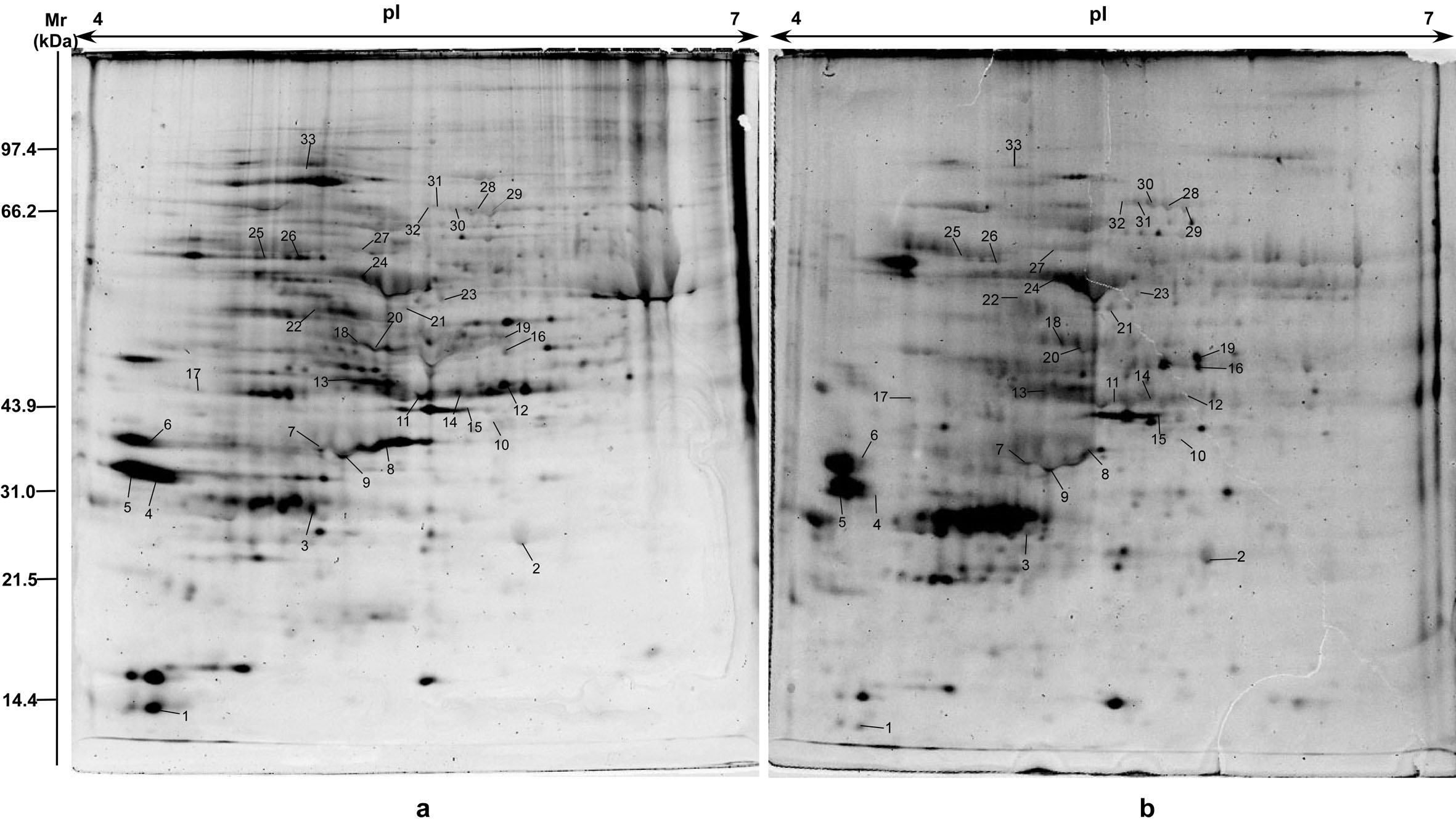


**Supplemental Figure S4**


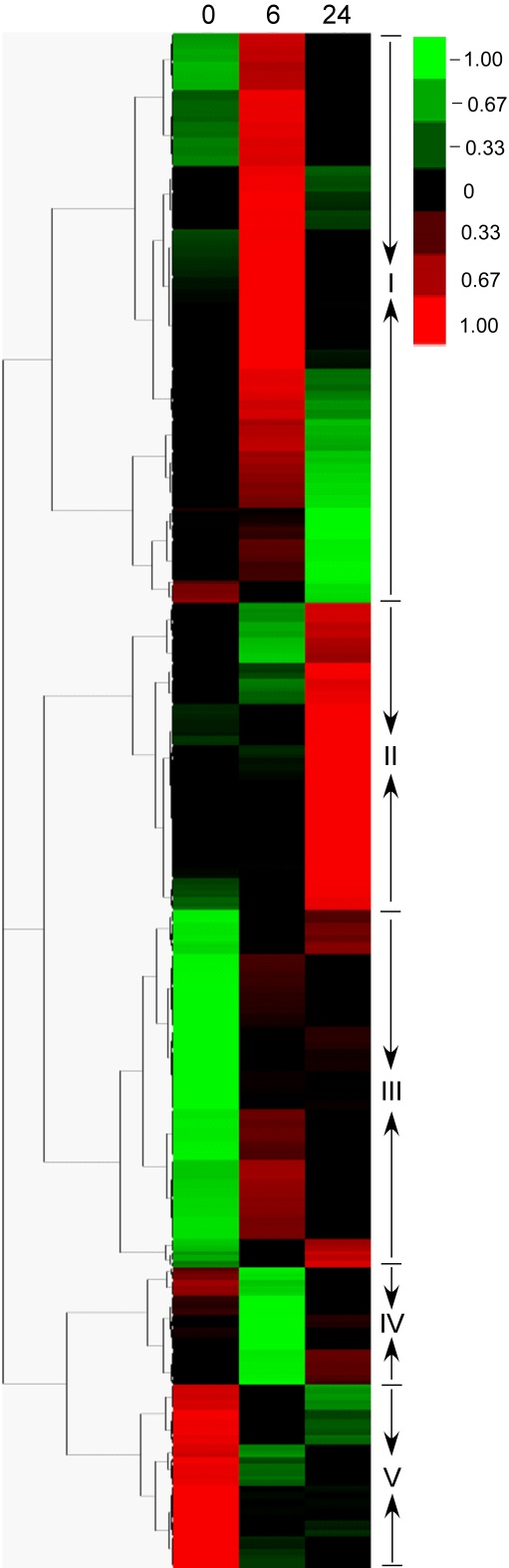


**Supplemental Figure S5**


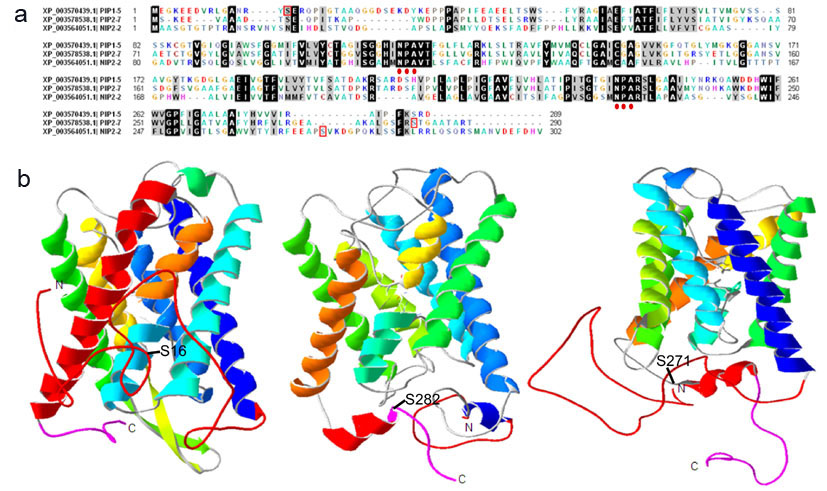


**Supplemental Figure S6**


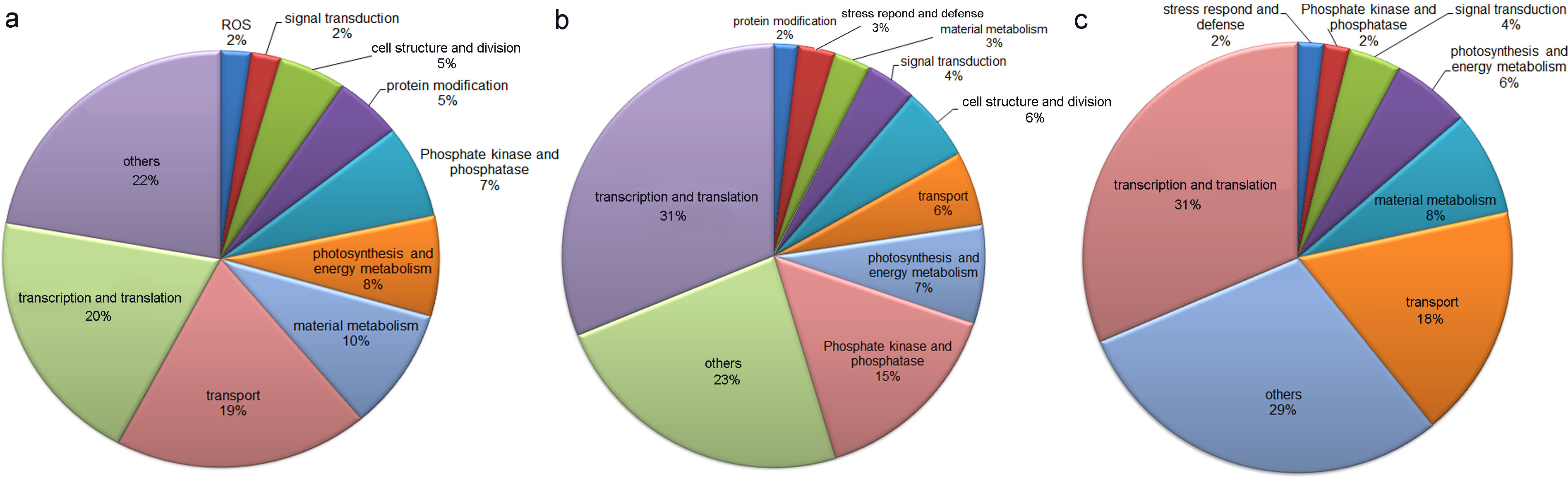


**Supplemental Figure S7**

**
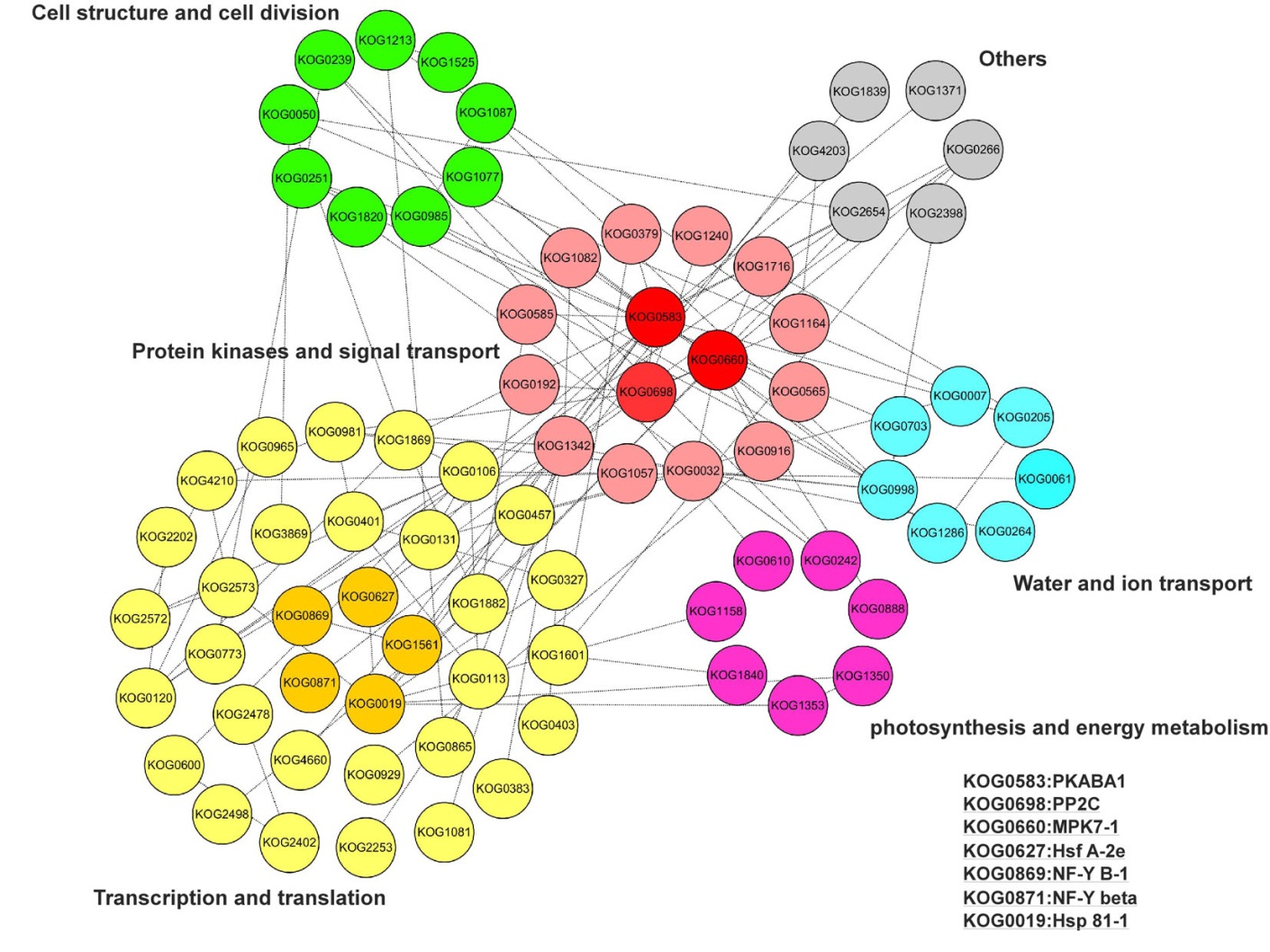
**

**Supplemental Figure S8**
